# Disturbance of the let-7/LIN28 double-negative feedback loop is associated with radio- and chemo-resistance in non-small cell lung cancer

**DOI:** 10.1371/journal.pone.0172787

**Published:** 2017-02-24

**Authors:** Jun Yin, Jian Zhao, Weimin Hu, Guangping Yang, Hui Yu, Ruihao Wang, Linjing Wang, Guoqian Zhang, Wenfan Fu, Lu Dai, Wanzhen Li, Boyu Liao, Shuxu Zhang

**Affiliations:** 1 Department of Radiotherapy, Affiliated Cancer Hospital & Institute of Guangzhou Medical University, Guangzhou, Guangdong, China; 2 Department of Chest Surgery, Affiliated Cancer Hospital & Institute of Guangzhou Medical University, Guangzhou, Guangdong, China; 3 Department of Abdominal Surgery, Affiliated Cancer Hospital & Institute of Guangzhou Medical University, Guangzhou, Guangdong, China; University of South Alabama Mitchell Cancer Institute, UNITED STATES

## Abstract

Radio- and chemo-resistance represent major obstacles in the therapy of non-small-cell lung cancer (NSCLC) and the underlying molecular mechanisms are not known. In the present study, during induction of radio- or chemo-resistance in NSCLC cells, dynamic analyses revealed that decreased expression of let-7 induced by irradiation or cisplatin resulted in increased expression of its target gene *LIN28*, and increased expression of LIN28 then contributed to further decreased expression of let-7 by inhibiting its maturation and biogenesis. Moreover, we showed that down-regulation of let-7 and up-regulation of LIN28 expression promoted resistance to irradiation or cisplatin by regulating the single-cell proliferative capability of NSCLC cells. Consequently, in NSCLC cells, let-7 and LIN28 can form a double-negative feedback loop through mutual inhibition, and disturbance of the let-7/LIN28 double-negative feedback loop induced by irradiation or chemotherapeutic drugs can result in radio- and chemo-resistance. In addition, low expression of let-7 and high expression of LIN28 in NSCLC patients was associated significantly with resistance to radiotherapy or chemotherapy. Therefore, our study demonstrated that disturbance of the let-7/LIN28 double-negative feedback loop is involved in the regulation of radio- and chemo-resistance, and that let-7 and LIN28 could be employed as predictive biomarkers of response to radiotherapy or chemotherapy in NSCLC patients.

## Introduction

Lung cancer is the leading cause of cancer-related death worldwide. Lung cancer can be divided into two major histologic subtypes: non-small-cell lung cancer (NSCLC) and small-cell lung cancer (SCLC). Approximately 80% of lung-cancer cases are NSCLC. Due to a lack of effective means for early screening and diagnosis, ≈70%–80% of NSCLC cases are diagnosed at an advanced stage and, thus, are inoperable. Therefore, radiotherapy and chemotherapy are the two principal treatments for NSCLC [1, 2]. However, resistance to irradiation or chemotherapeutic drugs can result in low rates for cure and treatment failure. The molecular mechanisms of radio-resistance and chemo-resistance are understood incompletely [3–5], so, research in these areas is important for successful therapy of NSCLC.

MicroRNA (miRNA) is highly conserved and highly specific in tissue. miRNA is an important endogenous non-coding small molecule RNA of length ≈18–25 nucleotides [6]. At present, >1000 miRNAs have been identified in humans. These miRNAs may regulate expression of ≥30% of human genes. Moreover, miRNA-mediated silencing of gene expression is important in many physiologic and pathologic processes (e.g., differentiation, proliferation, apoptosis) and several human diseases (e.g., cancer) [7–11]. Recently, dysregulation of miRNAs has also been observed during radiotherapy or chemotherapy [12–16]. In NSCLC, many miRNAs, such as miR-17, miR-92, and let-7, have abnormal regulation and are associated with the response to radiotherapy or chemotherapy, suggesting that miRNAs have a close relationship with the molecular mechanisms of radio- and chemo-resistance [17–20].

In the present study, we found that let-7 expression decreased in irradiation-resistant A549/IR cells and cisplatin-resistant A549/DDP cells compared with radio- and chemo-sensitive A549 cells. Furthermore, decreased expression of let-7 resulted in increased expression of the target gene LIN28, which was first identified in *Caenorhabditis elegans* as an important regulator of developmental timing (lin-28) [21]. In humans, two homologs of *lin-28*, *LIN28A* and *LIN28B* (which can bind to the terminal loops of the precursors of the let-7 family and block their processing into mature miRNAs) have important roles in human stem cells [21]. In NSCLC cells, we also found that LIN28 regulated let-7 expression negatively by binding to let-7 precursors and blocking their maturation. In addition, down-regulation of let-7 expression and up-regulation of LIN28 expression increased the colony-formation capacity of NSCLC cells. Consequently, disturbance of the let-7/LIN28 double-negative feedback loop induced by irradiation or chemotherapeutic drugs was related closely to the radio- and chemo-resistance of NSCLC cells. Moreover, expression of let-7 and LIN28 in NSCLC tissues was associated with the response to radiotherapy or chemotherapy. These findings suggest that a let-7/LIN28 double-negative regulatory loop is involved in the regulation of radio- and chemo-resistance, and that let-7 and LIN28 are potential new biomarkers for the response to radiotherapy or chemotherapy in NSCLC.

## Materials and methods

### Ethical approval of the study protocol

NSCLC tissues were collected from the Cancer Center of Guangzhou Medical University (Guangzhou, China) with written informed consent and permission from the Institutional Review Board. All patients provided written informed consent. The study protocol was approved by the ethics committee of Cancer Center of Guangzhou Medical University (approval number (2014) 66).

### Cell lines and cell culture

Human non-small cell lung cancer cells A549 (parental), A549/IR (irradiation-resistant) and A549/DDP (cisplatin-resistant) were cultured in RPMI 1640 medium containing 10% fetal bovine serum at 37°C. A549/IR cells were induced. Briefly, A549 cells were treated with 2 Gy of irradiation using a linear accelerator (Primart 6 MV; Siemens, Germany) and returned to the incubator. After 2 days, cells were irradiated again (second fraction). Fractionated irradiations were continued until the total dose reached 30 Gy. A549/DDP cells were induced using increasing concentrations of cisplatin, as described previously [19, 20]. A549/IR cells were treated with 2 Gy of irradiation once a week. A549/DDP cells were cultured with 2 μg/mL cisplatin.

### Collection of tissue samples

Sixty-nine NSCLC patients were recruited into this study. Inclusion criteria were patients: with primary NSCLC; with a histologic diagnosis of NSCLC with at least one measurable lesion; with a TNM clinical stage of IIIB to IV; who had undergone radiotherapy or first-line chemotherapy with platinum-based drugs. As described in detail previously [19], tissue samples were obtained and divided into two groups according to patient responses assessed using Response Evaluation Criteria in Solid Tumors (RECIST). That is, patients with a response or partial response to treatment were considered to be “treatment-sensitive” (R, responder), and patients with stable or progressive disease were considered to be “treatment-resistant” (NR, non-responder).

### Microarray detection of miRNA expression

A microarray assay and data analyses were carried out as described previously [19, 20]. Briefly, total RNA from A549/IR cells, A549/DDP cells and A549 cells was isolated using a Total RNA Purification kit (Qiagen, Germany). Microarray hybridization assays were carried out in two experiments: A549/IR cells (Cy5-labeled) compared with A549 cells (Cy3-labeled), and A549/DDP cells (Cy5-labeled) compared with A549 cells (Cy3-labeled). Hybridization images were collected using a laser scanner and analyzed by (i) subtracting the background and (ii) normalizing signals using a LOWESS filter.

### RNA extraction and quantitative reverse transcription-polymerase chain reaction (qRT-PCR)

A RT-PCR assay and data analyses were carried out, as described previously [19, 20]. Briefly, total RNA in cells and tissue samples was extracted by TriZOL^™^ (Invitrogen, USA). For detection of mRNA, mature miRNA, and primary miRNA, RT-PCR was done using gene specific primers or the miScript^™^ Primer Assay kit (Qiagen) after reverse transcription from RNA to cDNA. Relative expression of mRNA and miRNA was normalized by β-actin and U6 (Sigma–Aldrich, USA), respectively. mRNA RT-PCR primers (forward and reverse, respectively) were designed: *LIN28A*, 5′-GGAAAGAGCATGCAGAAGCG-3′ and 5′-GAATAGCCCCCACCCATTGT-3′; *LIN28B*, 5′-TTGACAAAGTCACGTGTGCTC-3′ and 5′-CCTCAGCTCCAAACTCGTGA-3′; *β-actin*, 5′-AGCGAGCATCCCCCAAAGTT-3′ and 5′-GGGCACGAAGGCTCATCATT-3′.

### Luciferase activity of cells

Dual luciferase analyses and data analyses were carried out as described previously [19, 20]. Briefly, the wild-type (WT) and mutant-type (MUT) 3′ untranslated region (3′-UTR) of LIN28 were synthesized chemically and inserted into the psiCHECK^™^-2 vector (Promega, USA) to obtain psiCHECK^™^-2-LIN28-3′-UTR (WT or MUT plasmids). Cells were transfected with WT plasmids or MUT plasmids in the presence of miRNA mimics or a non-target control (NC). Forty-eight hours after transfection, the luciferase activity of cells was measured.

### Cell transfection

Cells were transfected with 50 nM of miRNA mimics/inhibitors, siRNAs/plasmids or NCs by Lipofectamine Transfection Reagent according to manufacturer instructions (Invitrogen). Here, we used a mimics mixture of eight let-7 members and an inhibitors mixture of eight let-7 members to enhance and inhibit all eight members of let-7 family simultaneously (Qiagen).

### Western blotting

Western blotting was done as described previously [19, 20]. Briefly, proteins were extracted from cells transfected with 50 nM of miRNA mimics/inhibitors or NCs for SDS–PAGE analyses. The first antibody was rabbit polyclonal anti-LIN28A and anti-LIN28B (1:1000 dilution; Cell Signaling, USA). The secondary antibody was goat-anti-rabbit IgG conjugated with horseradish peroxidase at 1:1000 dilution. β-actin was used as an internal control.

### Cell cytotoxicity assays

A cell cytotoxicity assay and data analyses were carried out as described previously [19, 20]. Briefly, cell suspensions, which were transfected with 50 nM of miRNA mimics/inhibitors, siRNAs/plasmids or NCs, were exposed to different doses of irradiation or incubated in different concentrations of cisplatin. After 48 h, irradiation- or cisplatin-induced cytotoxicity was determined using CCK8 (Dojindo Laboratories, Japan) and represented as ED_50_ (Gy) or IC_50_ (μg/ml), respectively.

### Colony-formation assay

Cells were transfected with 50 nM of miRNA mimics/inhibitors, siRNAs/plasmids or NCs in 60-mm Petri dishes. Twenty-four hours after transfection, the cells were treated with irradiation (2 Gy) or cisplatin (2 μg/ml). After 24 h, cells were plated in six-well plates (1000 cells per well) and allowed to form colonies over 7 days. Then, cells were stained with GIEMSA and counted using ImageJ software.

### Statistical analyses

Values are the mean ± standard deviation (SD) of at least three separate experiments. The ED_50_ and IC values were assessed by linear regression analysis. The Student’s unpaired *t*-test, Mann–Whitney *U*-test, chi-square test, log-rank statistic, Spearman's rank correlation tests were done, and Receiver Operating Characteristic (ROC) curves created, using SPSS v21.0 (IBM, USA). *P* < 0.05 (two-tailed) was considered significant.

## Results

### Let-7 expression is down-regulated by irradiation or cisplatin treatment

**Fig 1:**
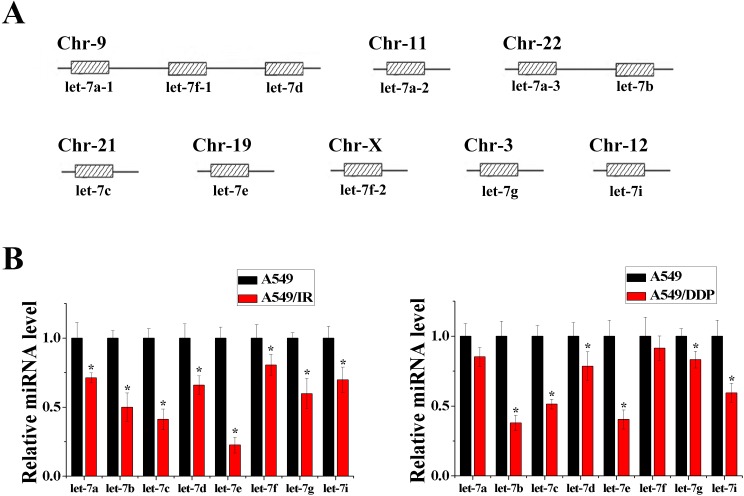
Expression of let-7 family miRNAs was down-regulated in radio- or chemo-resistant cells. **(A)** Members of the let-7 family (schematic). **(B)** Down-regulation of expression of let-7 family miRNAs in microarray experiments was validated by RT-PCR (n = 3, *P < 0.05).

We compared the miRNA expression of irradiation-resistant A549/IR cells or cisplatin-resistant A549/DDP cells with parent A549 cells using miRNA microarray analyses. Of note, compared with A549 cells, expression of eight members of the let-7 family (let-7a, let-7b, let-7c, let-7d, let-7e, let-7f, let-7g, let-7i) was down-regulated to varying degrees in A549/IR cells and A549/DDP cells (S1 and S2 Tables), though these miRNAs were distributed in different chromosomes (Fig 1A). Consistent with the results of microarray analyses, RT-PCR results confirmed reduced expression of these eight miRNAs in A549/IR cells or A549/DDP cells compared with A549 cells (Fig 1B). These findings suggested that the let-7 family is closely associated with radio- or chemo-resistance in NSCLC.

### Let-7 confers radio- and chemo-resistance in A549 cells by targeting LIN28

**Fig 2:**
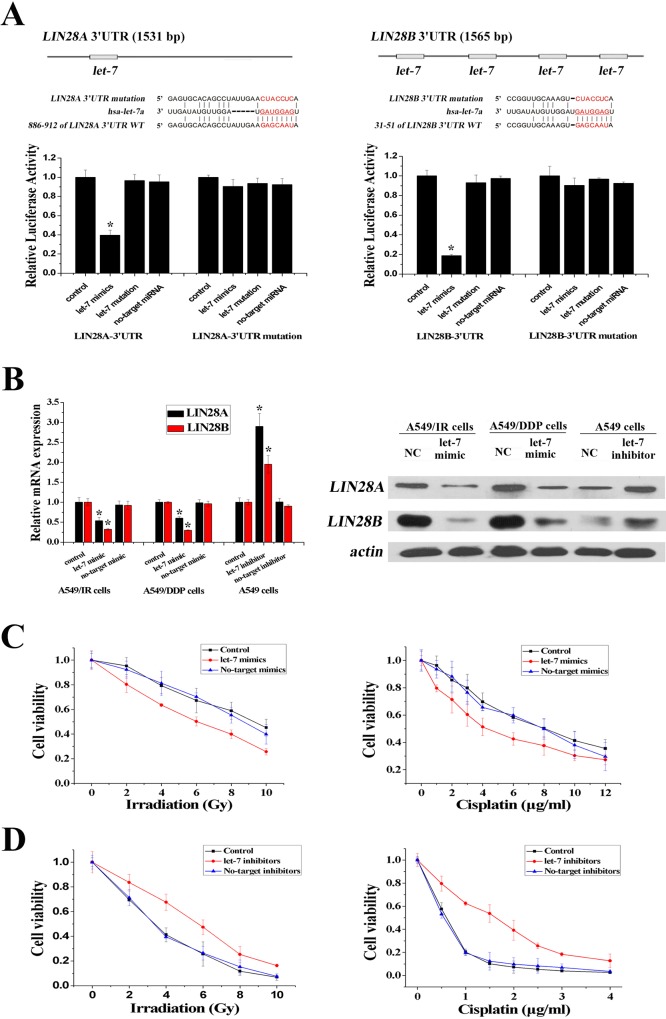
Let-7 family miRNAs regulated radio- and chemo-resistance by targeting *LIN28*. **(A)** Luciferase assays of let-7 targeting effects on *LIN28*. Mutations were generated in the *LIN28A* 3′-UTR and *LIN28B* 3′-UTR sequence in the complementary site for the seed region of let-7 as indicated. **(B)** mRNA and protein levels of LIN28 were decreased in A549/IR and A549/DDP cells after transfection of let-7 mimics and increased in A549 cells after transfection of let-7 inhibitors, as detected by RT-PCR and western blotting. **(C)** Overexpression of let-7 decreased resistance to irradiation in A549/IR cells or to cisplatin in A549/DDP cells significantly. **(D)** Inhibition of let-7 increased resistance to irradiation or cisplatin in A549 cells significantly (n = 3, *P < 0.05).

According to similarities in the six-nucleotide seed sequence in the let-7 family, eight members of this family can regulate the same genes. By consulting the miRNA database TargetScan, *LIN28A* and *LIN28B* (two homologs of *lin-28*) were found to be potential target genes of the let-7 family and were selected for further identification. The 3′-UTR of *LIN28A* contains one let-7 binding site, and the 3′-UTR of *LIN28B* contains four let-7 binding sites (Fig 2A). To test the gene-silencing effects of let-7 on LIN28, we designed let-7 mimics (mixture of eight let-7 member mimics) and let-7 inhibitors (mixture of eight let-7 member inhibitors) to enhance and inhibit these eight let-7 members simultaneously. We also designed luciferase reporter plasmids containing the let-7 target site of *LIN28* 3′-UTR (WT plasmids) or a site-directed mutagenesis version (MUT plasmids). The dual-luciferase reporter assay showed that the let-7 mimics reduced the relative luciferase activity of *LIN28A* and *LIN28B* WT plasmids compared with MUT plasmids (Fig 2A). Moreover, expression of LIN28A and LIN28B, which was higher in A549/IR and A549/DDP cells compared with A549 cells, was decreased significantly in A549/IR and A549/DDP cells after let-7 mimics were added, and increased significantly in A549 cells after let-7 inhibitors were added (Fig 2B). These results suggested that *LIN28A* and *LIN28B* were the direct targets of the let-7 family.

To confirm the regulatory functions of let-7 on radio- and chemo-resistance, we altered the let-7 levels in A549/IR, A549/DDP and A549 cells by introducing let-7 mimics or inhibitors, respectively. As shown by the growth inhibition curve, up-regulation of let-7 expression restored sensitivity to irradiation in A549/IR cells and to cisplatin in A549/DDP cells (the ED_50_ decreased from 9.71 ± 0.12 to 6.17 ± 0.19 Gy, and the IC_50_ decreased from 8.82 ± 0.36 to 4.58 ± 0.29 μg/ml) (Fig 2C). Conversely, down-regulation of let-7 expression protected A549 cells from irradiation or cisplatin treatment (the ED_50_ increased from 3.27 ± 0.11 to 5.73 ± 0.08 Gy, and the IC_50_ increased from 0.59 ± 0.03 to 1.86 ± 0.18 μg/ml) (Fig 2D). Similar results were obtained in another NSCLC cell line H1299 (S1 Fig). Therefore, it was hypothesized that let-7 may play an important part in the radio- and chemo-resistance of NSCLC.

### LIN28 blocks the processing of primary let-7 and regulates radio- and chemo-resistance

**Fig 3:**
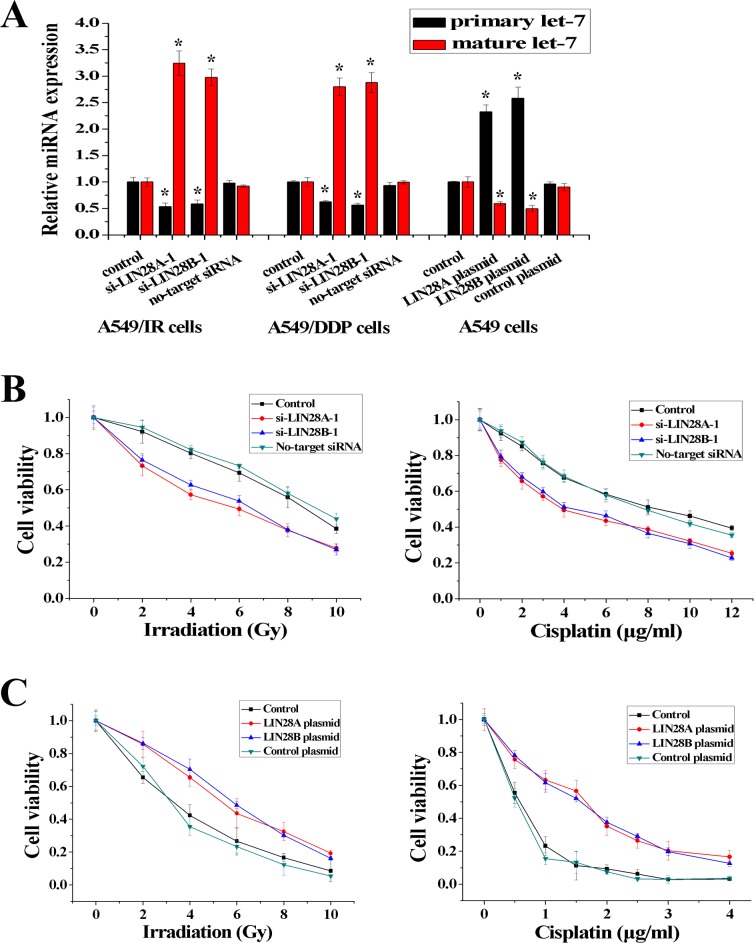
LIN28 blocked let-7 maturation specifically. **(A)** si-LIN28 increased the maturation of let-7 in A549/IR and A549/DDP cells and LIN28 plasmid decreased the maturation of let-7 in A549 cells, respectively. **(B)** Inhibition of LIN28 decreased resistance to irradiation in A549/IR cells or to cisplatin in A549/DDP cells significantly. **(C)** Overexpression of LIN28 increased resistance to irradiation or cisplatin in A549 cells significantly (n = 3, *P < 0.05).

It has been reported that LIN28 can block the maturation of let-7 [21]. To investigate the potential regulation of let-7 maturation by LIN28A and LIN28B in NSCLC cells, A549/IR and A549/DDP cells were transfected with two different siRNA (si-LIN28A-1 and si-LIN28A-2, si-LIN28B-1 and si-LIN28B-2) and A549 cells were transfected with expression plasmids (LIN28A plasmid, LIN28B plasmid) (S2 Fig). Knockdown of LIN28A or LIN28B resulted in a decrease in primary let-7 (pri-let-7) expression and an increase in mature let-7 expression in A549/IR and A549/DDP cells (Fig 3A). In contrast, overexpression of LIN28A or LIN28B increased levels of pri-let-7 and decreased levels of mature let-7 in A549 cells (Fig 3A and S2 Fig). We also investigated the effect of LIN28 on radio- and chemo-resistance of NSCLC cells. As shown in Fig 3B and S2 Fig, the resistance capacity of A549/IR or A549/DDP cells to irradiation or cisplatin decreased after LIN28A or LIN28B was inhibited (the ED_50_ decreased from 8.53 ± 0.27 to 6.15 ± 0.31 Gy, and the IC_50_ decreased from 7.92 ± 0.26 to 4.16 ± 0.19 μg/ml). In addition, the resistance capacity of A549 cells to irradiation or cisplatin increased after LIN28A or LIN28B was overexpressed (the ED_50_ increased from 3.36 ± 0.26 to 5.89 ± 0.17 Gy, and the IC_50_ increased from 0.55 ± 0.07 to 1.66 ± 0.13 μg/ml) (Fig 3C). Similar results were obtained in another NSCLC cell line H1299 (S1 Fig). These findings indicated that LIN28A and LIN28B may also have important roles in the radio- and chemo-resistance of NSCLC.

### The let-7/LIN28 double-negative feedback loop regulates radio- and chemo-resistance by controlling single-cell proliferative capability

**Fig 4:**
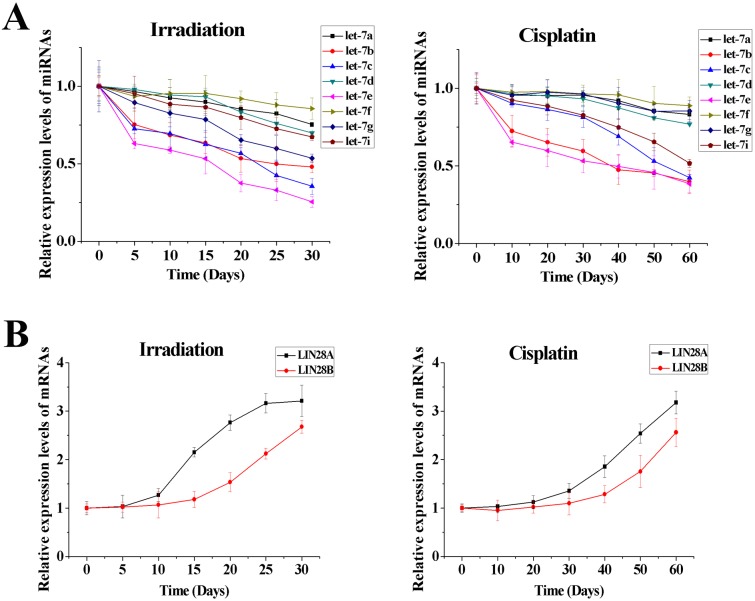
Dynamic analyses of expression of let-7 and LIN28 during induction of radio- or chemo-resistance in A549 cells. **(A)** To varying degrees, expression of let-7 miRNAs was down-regulated during induction of radio- or chemo-resistance. **(B)** To varying degrees, expression of LIN28A and LIN28B was up-regulated during induction of radio- or chemo-resistance.

We used RT-PCR to dynamically analyze the expression of eight members of the let-7 family (let-7a, let-7b, let-7c, let-7d, let-7e, let-7f, let-7g, let-7i) and two homologs of *lin-28* (LIN8A and LIN28B) during induction of radio- or chemo-resistance. We found that expression of all let-7 miRNAs decreased as A549 cells became increasingly resistant to irradiation or cisplatin (Fig 4A). During this process, expression of several let-7 miRNAs (let-7b, let-7c, let-7e in A549/IR cells and let-7b, let-7e in A549/DDP cells) decreased very rapidly and expression of the others decreased slowly. Down-regulation of let-7 could inhibit LIN28 expression, so we found that expression of LIN28A and LIN28B increased during this process, and LIN28A reached a peak more quickly (Fig 4B). As mentioned above, LIN28, identified as the direct target of let-7, was responsible for the suppression of let-7 maturation. Thus, let-7 and LIN28 negatively regulated each other and formed the let-7/LIN28 double-negative feedback loop during induction of radio- or chemo-resistance in NSCLC cells. In addition, the increase in LIN28A/B lagged behind the decrease in some let-7 miRNAs that decreased rapidly, suggesting that the rapid decrease in let-7 miRNAs resulted in an increase in LIN28A/B and then all let-7 miRNAs were inhibited further by increased LIN28A/B. Therefore, it is suggested that the down-regulation of let-7 expression induced by irradiation or cisplatin resulted in disturbance of the let-7/LIN28 double-negative feedback loop, which may be associated with radio- and chemo-resistance in NSCLC.

**Fig 5:**
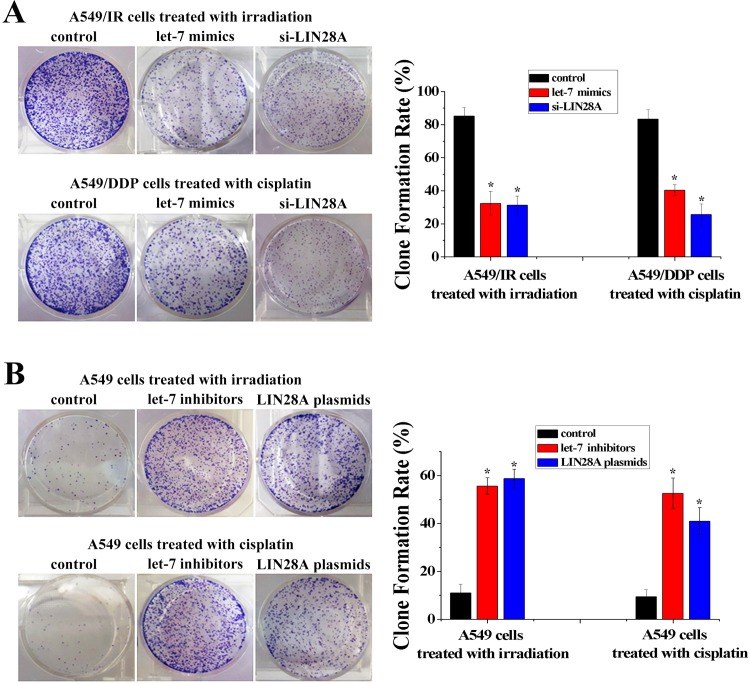
Let-7 and LIN28 were putative regulators of single-cell proliferative capability. **(A)** Colony-formation assay of A549/IR and A549/DDP cells transfected with let-7 mimics or si-LIN28A. **(B)** Colony-formation assay of A549 cells transfected with let-7 inhibitors or LIN28A plasmids (n = 3, *P < 0.05).

Given the disturbance in the let-7/LIN28 double-negative feedback loop identified in A549/IR and A549/DDP cells, we wondered how this regulatory loop influenced the radio- and chemo-resistance of NSCLC. let-7 and LIN28 have been found to be associated with cancer stem cells, so we investigated the effect of let-7 and LIN28 on the single-cell proliferative capability of NSCLC cells using a colony-formation assay. In A549/IR cells treated with irradiation or A549/DDP cells treated with cisplatin, the colony-formation capacity was decreased significantly after let-7 mimics or siRNA-LIN28 had been transfected (Fig 5A). In contrast, in A549 cells treated with irradiation or cisplatin, the colony-formation capacity was increased significantly after let-7 inhibitors or LIN28 expression plasmids had been transfected (Fig 5B). Similar results were obtained in another NSCLC cell line H1299 (S1 Fig). Thus, these data indicated that disturbance of the let-7/LIN28 double-negative feedback loop induces radio- and chemo-resistance by regulating the single-cell proliferative capability of NSCLC cells.

### Let-7 and LIN28 as potential prognostic biomarkers of treatment outcome after radiotherapy or chemotherapy in NSCLC

**Fig 6:**
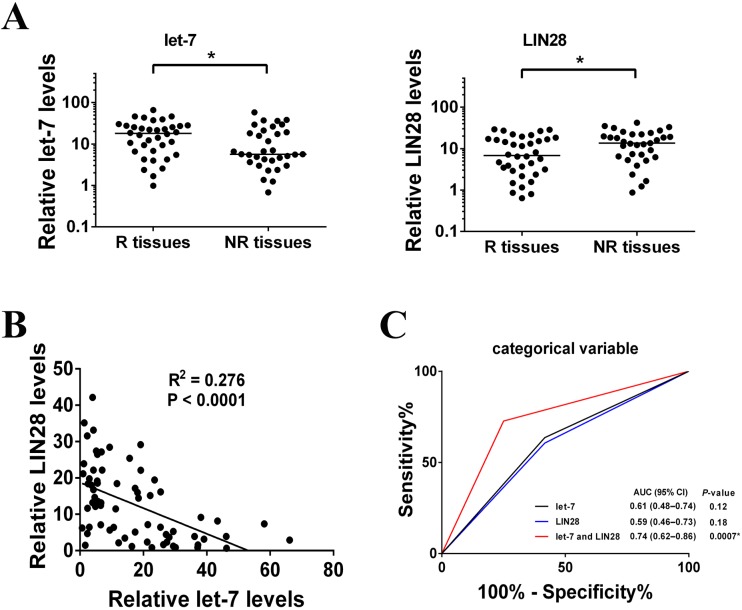
Expression of let-7 and LIN28 was associated with radio- and chemotherapy response. **(A)** Scatter plots of expression of let-7 and LIN28 in responders and non-responders (R, responder (response or partial response); NR, non-responder (stable or progressive disease)). (*P < 0.05) **(B)** Correlation between the relative RNA expression of let-7 and LIN28 in specimens from 69 patients with NSCLC, expressed using the square of Spearman’s correlation coefficients (R^2^) and linear regression (solid lines). Significance was calculated by Spearman's rank correlation test. **(C)** ROC analyses assessing the association of let-7 and LIN28 and radio- and chemotherapy response (NR or R) by combinations of let-7 and LIN28 levels using the sum of scores as categorical variables, where let-7 and LIN28 were dichotomized and their categories represented by the score of 0 or 1 as follows: score 0 (low risk) = let-7 or LIN28 levels ≥ median; score 1 (high risk) = converse of criteria for score 0.

The let-7/LIN28 double-negative feedback loop was found to be associated with radio- and chemo-resistance in NSCLC cells, so we wondered if the let-7/LIN28 loop may be associated with the outcome of radiotherapy or chemotherapy in NSCLC patients. Here, we ignored the regulatory differences in members of the let-7 family or homologs of *lin-28*, and used mean expression values to represent the expression of let-7 and LIN28. Using RT–PCR, endogenous expression of let-7 was found to be significantly lower in radio- or chemo-resistant NSCLC tissues (NR tissues) compared with radio- or chemo-sensitive NSCLC tissues (R tissues) (Fig 6A). In contrast to let-7, endogenous expression of LIN28 was significantly higher in NR tissues than in R tissues (Fig 6A). These results indicated that low expression of let-7 and high expression levels of LIN28 were positively correlated with the poor effect of radiotherapy or chemotherapy in NSCLC patients. These significant inverse correlations were observed when LIN28 expression was plotted against let-7 expression (r = − 0.525, P < 0.0001) (Fig 6B), suggesting that let-7 and LIN28 also negatively regulated each other *in vivo*. To determine the potential of let-7 and LIN28A as biomarkers, expression of let-7 and LIN28 was analyzed as categorical variables in ROC analyses. Compared with separate analyses of let-7 and LIN28, combined analyses of let-7 and LIN28 produced a higher area under the curve (AUC) score of 0.74 (95% CI, 0.62–0.86) (Fig 6C), indicating that let-7 and LIN28 could be used as potential biomarkers for predicting the clinical outcome of radiotherapy or chemotherapy in NSCLC.

## Discussion

Radiotherapy and chemotherapy are very important in NSCLC treatment, radio- and chemo-resistance are obstacles to successful treatment [22]. Therefore, identification of the molecular mechanisms of radio- and chemo-resistance has long been a goal in cancer research.

Recently, it was found that abnormal expression of several miRNAs can result in radio- and chemo-resistance by deregulating resistant-associated cellular functions such as drug absorption, as well as the proliferation, apoptosis, and DNA repair of cells [23]. In the present study, decreased expression of let-7 was found in irradiation-resistant A549/IR cells, cisplatin-resistant A549/DDP cells and irradiation-resistant H1299/IR cells compared with radio- and chemo-sensitive A549 cells and H1299 cells, suggesting that let-7 is involved in the regulation of radio- and chemo-resistance in NSCLC.

let-7, which was identified originally in *C*. *elegans*, is a highly conserved miRNA, and the role of let-7 as a tumor suppressor has been reported because its defects can result in over-proliferation and lack of terminal differentiation in human cancers [24–26]. Based on recent studies, let-7 has been found to have an important role in regulating multiple oncogenes, such as RAS, cMYC, and HMGA2 [27, 28]. In addition, LIN28 has been recognized as an oncogenic protein implicated in RNA binding and regulation of miRNA maturation [21]. Increased expression of LIN28 has been found to be associated with a poor prognosis because it facilitates cancer-cell metastasis and increases resistance to chemotherapeutic drugs in different human cancers [29, 30]. According to our results, down-regulation of let-7 expression increased LIN28 expression, which repressed the maturation and biogenesis of let-7 in NSCLC cells, indicating that a double-negative feedback loop was established between let-7 and LIN28 during the induction of radio- or chemo-resistance in NSCLC cells. The let-7/LIN28 regulatory loop is highly conserved and can operate as a “switch” to maintain differentiated or embryonic cell fates [31–33]. Here, it was also shown that the let-7/LIN28 regulatory loop affected radio- and chemo-resistance by regulating the single-cell proliferative capability of NSCLC cells. Moreover, let-7 and LIN28 were associated significantly with the response to radiotherapy or chemotherapy, and have the potential to be therapeutic response biomarkers in NSCLC.

As reported, it is of critical importance to strictly regulate the appropriate homeostatic status of the let-7/LIN28 double-negative feedback loop because slight changes in levels of let-7 or LIN28 can be amplified by the loop. This results in more significant alterations which are associated with the risk of developing human disease, such as cancers [25, 28]. As shown in the present study, during the induction of radio- or chemo-resistance in NSCLC cells, expression of some let-7 miRNAs (let-7b, let-7c and let-7e) decreased rapidly and some let-7 miRNAs (let-7a and let-7f) decreased slowly. Among members of the let-7 family, three copies of let-7a, two copies of let-7f and one copy of other members are located at different chromosomes in the human genome (Fig 1A) [24]. Therefore, it is suggested that the rapid decrease in let-7 miRNAs that have only one copy can occur easily due to the genomic instability induced by irradiation or cisplatin during radiotherapy or chemotherapy. However, let-7a and let-7f have two or three copies, so reductions in let-7a and let-7f are not rapid because the genomic instability of all copies does not occur simultaneously. Conversely, of the two homologs of *lin-28*, LIN28A increased more quickly than LIN28B during induction of radio- or chemo-resistance in NSCLC cells. According to sequencing analyses, the 3′-UTR of LIN28A contains only one let-7 binding site and the 3′-UTR of LIN28B contains four let-7 binding sites. Thus, LIN28A can escape the inhibition of let-7 more easily than LIN28B. Therefore, during induction of radio- or chemo-resistance in NSCLC, first, irradiation or chemotherapeutic drugs can induce genomic instability and result in a rapid decrease in expression of some let-7 miRNAs. Then, the rapid decrease in let-7 miRNAs can weaken suppression of LIN28 mRNA and result in increased expression of LIN28 which, in turn, can reduce the level of all let-7 miRNAs. Consequently, homeostasis of the let-7/LIN28 loop (high let-7 and low LIN28) is disturbed and, due to the amplifying effect of the let-7/LIN28 double-negative feedback loop, LIN28 expression increases and let-7 expression decreases until a new balance (low let-7 and high LIN28) is achieved. Finally, as mentioned above, the low expression of let-7 and high expression of LIN28 can result in radio- and chemo-resistance. Based on these findings, it is suggested that disturbance of the let-7/LIN28 regulatory loop can also act as a switch to change NSCLC cells from being sensitive to radiotherapy or chemotherapy to being resistant. Moreover, we hypothesize that other mechanisms (e.g., gene mutation) that can also change the expression of let-7 and/or LIN28, can also induce disturbance of the let-7/LIN28 double-negative feedback loop, which is associated with radio- and chemo-resistance in NSCLC.

## Conclusions

The present study focused on radio- and chemo-resistance in NSCLC. We demonstrated that irradiation or chemotherapeutic drugs disturbed the let-7/LIN28 double-negative feedback loop involved in the regulation of radio- and chemo-resistance. Thus, these findings provide novel insight into the molecular mechanisms of radio- and chemo-resistance, and also provide a promising strategy to predict and reverse radio- and chemo-resistance in the future.

## Supporting information

S1 Table Differential expression of let-7 family in A549/IR cells compared with A549 cells. (DOC)

S2 Table Differential expression of let-7 family in A549/DDP cells compared with A549 cells. (DOC)

S1 Fig Both let-7 and LIN28 regulated radio-resistance in H1299 cells. **(A)** Down-regulation of let-7 family miRNAs in irradiation-resistant H1299/IR cells compared with its parental H1299 cells. **(B)** Up-regulation of Lin28 in H1299/IR cells compared with H1299 cells. **(C)** Overexpression of let-7 significantly decreased resistance to irradiation in H1299/IR cells. **(D)** Inhibition of let-7 significantly increased resistance to irradiation in H1299 cells. **(E)** Inhibition of LIN28 significantly decreased resistance to irradiation in H1299/IR cells. **(F)** Overexpression of LIN28 significantly increased resistance to irradiation in H1299 cells. **(G)** Colony formation assay of H1299/IR cells transfected with let-7 mimics or si-LIN28A. Colony formation assay of H1299 cells transfected with let-7 inhibitors or LIN28A plasmids. (n = 3, *P < 0.05)
(TIF)

S2 Fig LIN28 regulated radio- and chemo-resistance. **(A)** The protein levels of LIN28 were decreased after transfection of si-LIN28 and increased after transfection of LIN28 plasmids, detected by western blotting assays. **(B)** si-LIN28 increased the maturation of let-7 in A549/IR and A549/DDP cells. **(C)** Inhibition of LIN28 significantly decreased resistance to irradiation in A549/IR cells or to cisplatin in A549/DDP cells. (n = 3, *P < 0.05)
(TIF)

S3 Fig Western Blotting. The full-sized, unadjusted and uncropped Western blot images.
(TIF)

S1 Dataset A549+A549_IR_Data. The miRNA expression profiles of A549 cells and A549/IR cells.
(XLSX)

S2 Dataset A549+A549_DDP_Data. The miRNA expression profiles of A549 cells and A549/DDP cells.
(XLSX)
