# Supplementary material for: Disturbance of the let-7/LIN28 double-negative feedback loop is associated with radio- and chemo-resistance in non-small cell lung cancer
Source: PLoS One. 2017 Feb 24;12(2):e0172787. doi: 10.1371/journal.pone.0172787 (PMC5325287; doi:10.1371/journal.pone.0172787)
Supplement: S1 Table — (DOC) [file pone.0172787.s001.doc]

**Table S1.** Differential expression of let-7 family in A549/IR cells compared with A549 cells.

| **miRNA ID** | **Signal Median (A549 cells)** | **Signal Median (A549/IR cells)** | **Fold Change (log2)** | ***P* Value** |
| --- | --- | --- | --- | --- |
| hsa-let-7a | 12995.84 | 10406.89 | -0.32 | 0.001 |
| hsa-let-7b | 4315.52 | 2415.59 | -0.84 | 0.005 |
| hsa-let-7c | 7491.12 | 4013.08 | -0.89 | 0.005 |
| hsa-let-7d | 8552.60 | 6608.00 | -0.38 | 0.024 |
| hsa-let-7e | 4250.14 | 1267.29 | -1.74 | 0.005 |
| hsa-let-7f | 11151.93 | 9285.73 | -0.27 | 0.033 |
| hsa-let-7g | 2952.71 | 2195.14 | -0.43 | 0.025 |
| hsa-let-7i | 2932.61 | 2440.40 | -0.27 | 0.069 |
