# Supplementary material for: Disturbance of the let-7/LIN28 double-negative feedback loop is associated with radio- and chemo-resistance in non-small cell lung cancer
Source: PLoS One. 2017 Feb 24;12(2):e0172787. doi: 10.1371/journal.pone.0172787 (PMC5325287; doi:10.1371/journal.pone.0172787)
Supplement: S2 Table — (DOC) [file pone.0172787.s002.doc]

**Table S2.** Differential expression of let-7 family in A549/DDP cells compared with A549 cells.

| **miRNA ID** | **Signal Median (A549 cells)** | **Signal Median (A549/DDP cells)** | **Fold Change (log2)** | ***P* Value** |
| --- | --- | --- | --- | --- |
| hsa-let-7a | 15401.40 | 14837.19 | -0.06 | 0.054 |
| hsa-let-7b | 8832.05 | 3797.79 | -1.22 | <0.001 |
| hsa-let-7c | 11735.44 | 7338.57 | -0.67 | 0.003 |
| hsa-let-7d | 10723.40 | 9604.47 | -0.15 | 0.007 |
| hsa-let-7e | 6122.12 | 2798.04 | -1.12 | <0.001 |
| hsa-let-7f | 14739.82 | 14724.42 | -0.01 | 0.962 |
| hsa-let-7g | 7103.27 | 6835.35 | -0.06 | 0.170 |
| hsa-let-7i | 12812.07 | 7493.22 | -0.79 | 0.014 |
