# Supplementary figures and images for: Disturbance of the let-7/LIN28 double-negative feedback loop is associated with radio- and chemo-resistance in non-small cell lung cancer
Source: PLoS One. 2017 Feb 24;12(2):e0172787. doi: 10.1371/journal.pone.0172787 (PMC5325287; doi:10.1371/journal.pone.0172787)

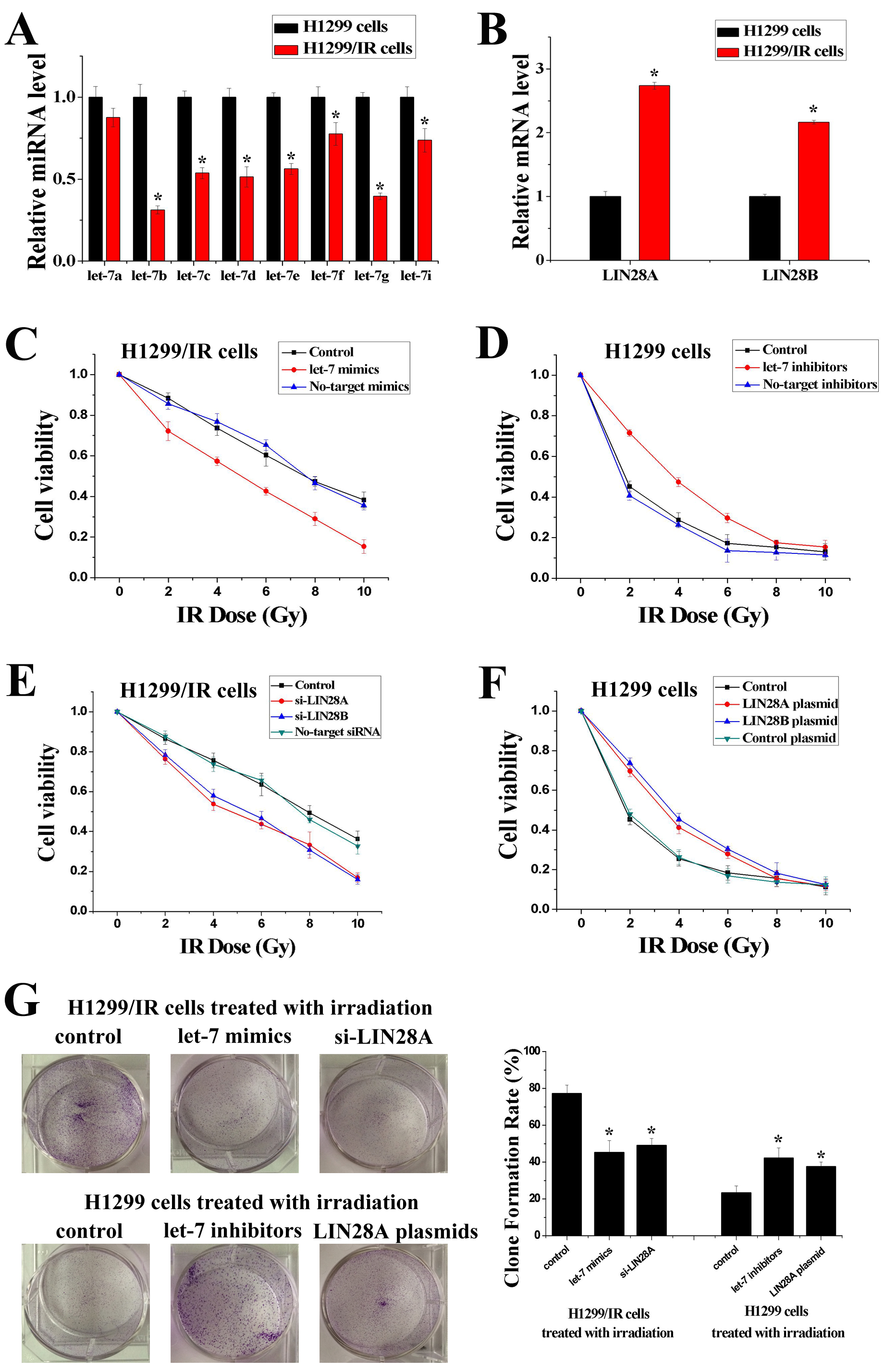

Supplement: S1 Fig — (A) Down-regulation of let-7 family miRNAs in irradiation-resistant H1299/IR cells compared with its parental H1299 cells. (B) Up-regulation of Lin28 in H1299/IR cells compared with H1299 cells. (C) Overexpression of let-7 significantly decreased resistance to irradiation in H1299/IR cells. (D) Inhibition of let-7 significantly increased resistance to irradiation in H1299 cells. (E) Inhibition of LIN28 significantly decreased resistance to irradiation in H1299/IR cells. (F) Overexpression of LIN28 significantly increased resistance to irradiation in H1299 cells. (G) Colony formation assay of H1299/IR cells transfected with let-7 mimics or si-LIN28A. Colony formation assay of H1299 cells transfected with let-7 inhibitors or LIN28A plasmids. (n = 3, *P < 0.05) (TIF) [file pone.0172787.s003.tif]

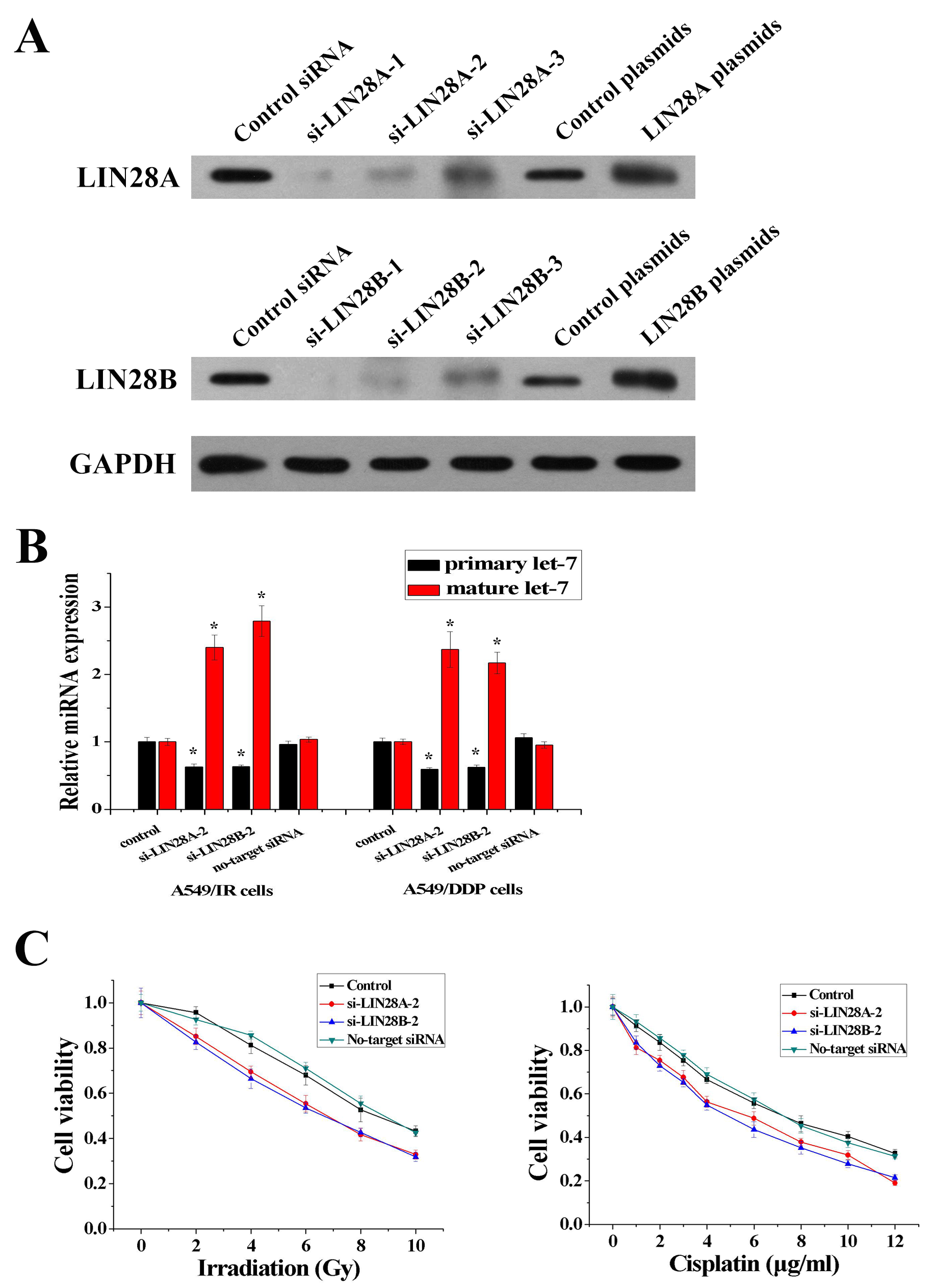

Supplement: S2 Fig — (A) The protein levels of LIN28 were decreased after transfection of si-LIN28 and increased after transfection of LIN28 plasmids, detected by western blotting assays. (B) si-LIN28 increased the maturation of let-7 in A549/IR and A549/DDP cells. (C) Inhibition of LIN28 significantly decreased resistance to irradiation in A549/IR cells or to cisplatin in A549/DDP cells. (n = 3, *P < 0.05) (TIF) [file pone.0172787.s004.tif]

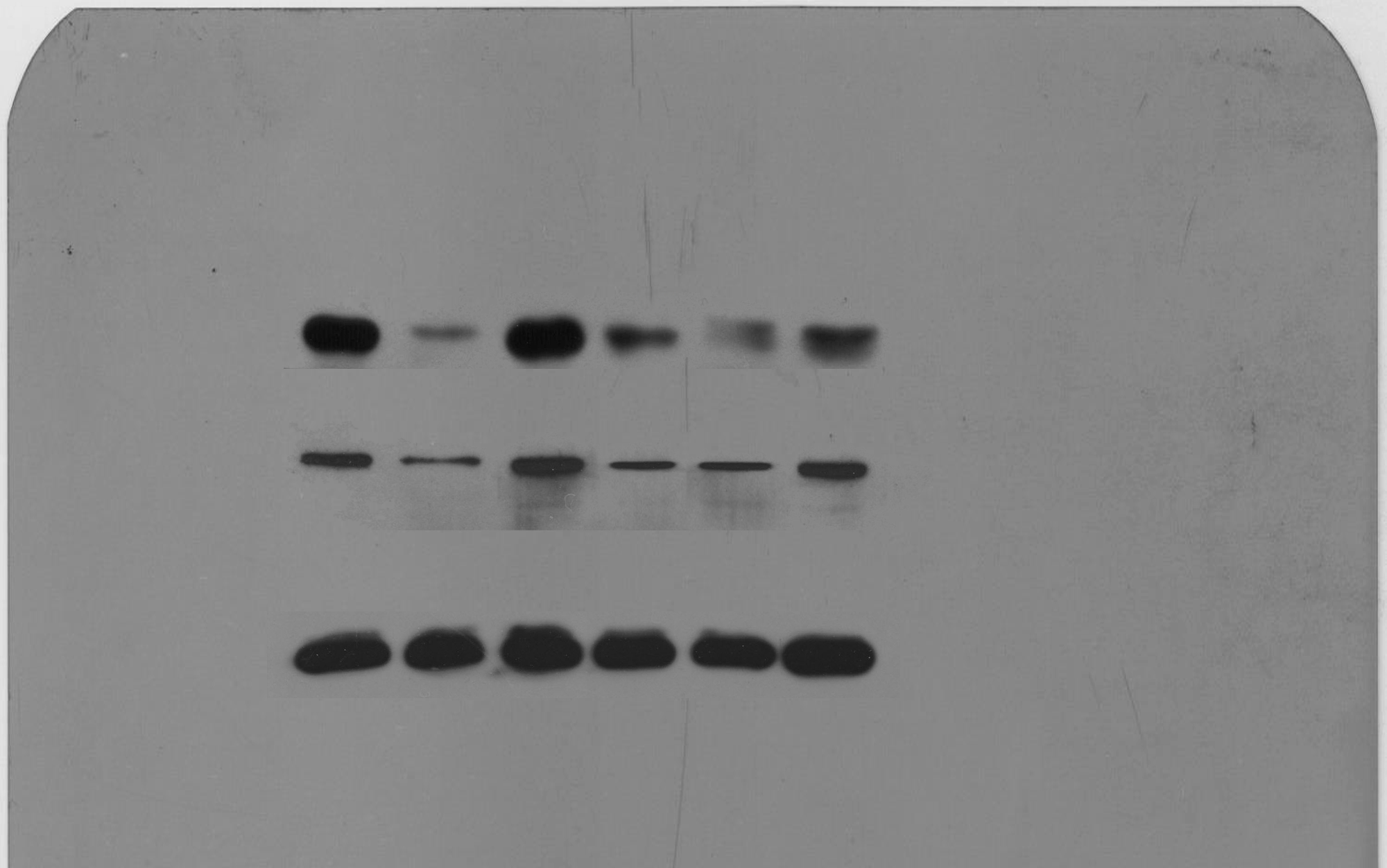

Supplement: S3 Fig — The full-sized, unadjusted and uncropped Western blot images. (TIF) [file pone.0172787.s005.tif]
